# The influence of exercise intentions on university students’ exercise behavior: the chain mediation of basic psychological needs and intrinsic motivation

**DOI:** 10.3389/fpsyg.2026.1873425

**Published:** 2026-09-23

**Authors:** Jinsong Luan

**Affiliations:** College of Exercise and Health, Shenyang Sport University, Shenyang, China

**Keywords:** basic psychological needs, exercise behavior, exercise intention, intrinsic motivation, university students

## Abstract

**Purpose:**

Based on self-determination theory, this study examines the effects of exercise intention, basic psychological needs, and intrinsic motivation on college students’ exercise behavior, and investigates the chain mediation effect of basic psychological needs on intrinsic motivation.

**Methods:**

A survey was conducted on 994 college students (mean age 20.64 ± 1.35) using the Exercise Intention Questionnaire, the Basic Psychological Needs Satisfaction Scale, the Exercise Behavior Regulation Questionnaire, and the Physical Activity Level Scale.

**Results:**

Exercise intention, basic psychological needs, and intrinsic motivation significantly were positively associated with exercise behavior [*F*(3,990) = 192.555, *P* < 0.001]. In the process by which exercise intention influences exercise behavior, the chain mediation effect of basic psychological needs–intrinsic motivation was significant (effect size 0.76, 95% CI [0.385, 1.155]).

**Conclusion:**

University students’ exercise intention, basic psychological needs, and intrinsic motivation can predict the occurrence of exercise behavior; exercise intention is directly linked to exercise behavior and is also associated with it through the chain mediation of basic psychological needs and intrinsic motivation.

## 1 Introduction

According to a World Health Organization report, physical inactivity has been identified as the fourth leading risk factor for mortality worldwide, following hypertension, tobacco use, and hyperglycemia. In many countries, levels of physical inactivity are rising, which has a significant impact on global health, the prevalence of noncommunicable diseases such as cardiovascular disease, diabetes, and cancer, and risk factors such as hypertension, hyperglycemia, and overweight. Research indicates that regular physical activity can reduce the risk of coronary heart disease, stroke, diabetes, hypertension, colorectal cancer, breast cancer, and depression. In May 2004, the 57th World Health Assembly adopted the Global Strategy on Diet, Physical Activity, and Health, recommending that Member States develop national action plans and policies on physical activity to increase physical activity levels among their populations (World Health Organization, 2004). In May 2008, the 61st World Health Assembly adopted the Global Strategy and the Global Action Plan for the Prevention and Control of Noncommunicable Diseases, recommending that adolescents aged 5 to 17 accumulate at least 60 min of moderate-to-vigorous intensity physical activity daily, with daily physical activity primarily consisting of aerobic exercise, and recommending exercise at least three times a week; It is recommended that adults aged 18–64 engage in at least 150 min of moderate-intensity aerobic exercise per week and perform muscle-strengthening activities involving major muscle groups on at least 2 days per week (World Health Organization, 2008). Regular physical activity can significantly improve adolescents’ health. Compared to physically inactive adolescents, those who are physically active exhibit higher levels of fitness, muscular endurance, muscle strength, and bone health, and experience reduced symptoms of anxiety and depression (World Health Organization, 2010).

Psychological research is dedicated to describing, explaining, and predicting behavior; therefore, the study of behavioral motivation has always been a focal point for psychologists. In the 1980s, American psychologists Edward Deci and Richard Ryan proposed Self-Determination Theory, emphasizing that humans possess a fundamental inclination toward self-determination. Self-regulation influences the generation of individual motivation, thereby guiding people to engage in activities that interest them and contribute to the development of their abilities (Bao and Zhang, 2005). Among these, the need for autonomy, the need for competence, and the need for relatedness are the three fundamental psychological needs of humans. The fulfillment of these basic psychological needs is a key factor in the generation of behavioral motivation, and the degree of motivation internalization is a function of the satisfaction of these needs. The need for autonomy refers to an individual’s ability to choose behaviors based on their own intentions, act entirely according to their own will, and gain a sense of autonomy through free choice; the need for competence refers to an individual’s assessment of their ability to complete a task and their capacity to handle tasks of varying difficulty, thereby gaining a sense of competence through task completion; the need for relatedness refers to people’s desire for mutual respect, support, understanding, and love from others during activities, thereby fulfilling their sense of belonging. To enhance intrinsic motivation or drive behavior by internalizing extrinsic motivation, the external environment must satisfy these three basic psychological needs. There is a dialectical relationship between the initiative of self-determination and the surrounding environment. When these three needs are generally met, they foster autonomous motivation in people, whereas a social environment that hinders the fulfillment of these needs reduces an individual’s autonomous motivation.

Self-determination theory emphasizes not only the influence of self-determination on behavior but also how self-determination ultimately affects behavior by influencing the level of motivation. Based on the intensity with which motivation drives behavior, motivation can be classified into intrinsic, extrinsic, and motivated. Among these, intrinsic motivation is the most autonomous; it involves individuals actively engaging in an activity to enjoy the pleasure of the activity or to gain a sense of fulfillment and achievement. External motivation stems from external conditions rather than the activity itself. Based on the degree of autonomy, external motivation can be further categorized from weak to strong as extrinsic regulation, introjective regulation, identification regulation, and integrative regulation. Extrinsic regulation refers to an individual participating in an activity to gain others’ approval, external rewards, or to avoid negative consequences; Internal regulation is driven by the desire to improve oneself or avoid feelings of guilt; identification regulation occurs when an individual perceives the activity as valuable or highly important; and integrative regulation emerges when an individual believes the activity is not only important to them but also aligns with their personal values and sense of self. When an individual’s level of self-determination is high, internal motivation is easily stimulated and sustained, thereby providing the driving force for behavior, which becomes more enduring.

Existing research has widely confirmed that the emergence of physical exercise behavior is closely related to Self-Determination Theory (SDT). The fulfillment of individuals’ basic psychological needs plays a decisive role in exercise behavior by influencing their motivation to exercise. (1) Basic Psychological Needs, Motivation, and Exercise Behavior: Mediating and Predictive Mechanisms. Research on adolescents also supports this view; a survey of 480 adolescents conducted by Cuevas-Campos et al. (2020) showed that motivation mediates the relationship between basic needs and the intention to engage in physical activity (Cuevas-Campos et al., 2020). Xue (2010) found that intrinsic motivation was significantly and positively correlated with the duration, intensity, frequency, and total volume of exercise (Xue, 2010). A survey by Fan (2018) of 803 college students also confirmed that basic psychological needs influence extracurricular physical exercise behavior by affecting self-determination and self-efficacy (Fan, 2018). Huang and Qiu (2019)’s study of 428 college students indicated that exercise motivation partially mediates the relationship between the satisfaction of basic psychological needs and exercise behavior (Huang and Qiu, 2019). Dong and Mao (2020)’s study of 2,052 college students further noted that identity, intrinsic motivation, and exercise commitment are all significant predictors of exercise behavior (Dong and Mao, 2020). Lu and Xi (2020)’s study of 518 college students delved even deeper, finding that basic psychological needs play different roles in the processes of motivation internalization and behavioral promotion, with the full mediating effect of the need for autonomy being the most significant (Lu and Xi, 2020).

(2) The Impact of Need Satisfaction on Mental Health and Exercise Outcomes: Whether basic psychological needs are met not only influences exercise behavior but is also directly linked to an individual’s mental health and exercise outcomes. Vansteenkiste and Ryan (2013) noted that the frustration of basic psychological needs has a negative impact on mental health, leading to decreased motivation levels and, consequently, impaired exercise performance. Studies by Vlachopoulos et al. (2013) confirmed that the degree of satisfaction of basic psychological needs is significantly correlated with exercise behavior, and that exercise dependence is positively correlated with need satisfaction (Vlachopoulos et al., 2013). Furthermore, based on a survey of 664 Chinese adolescents, Xiang and Ding (2014) found that the fulfillment of the three basic psychological needs—autonomy, competence, and relatedness—all positively predicted subjective vitality and served as mediators (Xiang and Ding, 2014). This suggests that fulfilling psychological needs is a key pathway to enhancing the exercise experience and promoting mental health.

(3) Environmental factors, particularly autonomy support, are key external conditions for promoting the internalization of extrinsic motivation and stimulating intrinsic motivation. A study by Ding and Mao (2014) involving 371 Chinese high school students showed that a socially supportive environment not only stimulates adolescents’ intrinsic motivation to exercise but also promotes the internalization of extrinsic motivation, thereby effectively increasing their participation (Ding and Mao, 2014). A study by Bu et al. (2017) involving 480 middle school students provided counterevidence to this, finding that a low sense of autonomy support leads to reduced levels of intrinsic motivation for sports among adolescents, which in turn results in avoidance behaviors toward physical exercise (Bu et al., 2017). In the field of competitive sports, Li (2017)’s study of 220 adolescent track and field athletes showed that the fulfillment of basic psychological needs significantly predicted their exercise motivation, and that need fulfillment mediated the relationship between coaches’ autonomy support and athletes’ intrinsic motivation (Li, 2017).

While existing literature has extensively documented the individual roles of basic psychological needs and intrinsic motivation in promoting exercise behavior, most studies have focused on direct effects or simple mediations. There is a notable gap in understanding how ‘exercise intention’ acts as an antecedent in this process. Specifically, it remains unclear whether exercise intention influences behavior directly or if it triggers a sequential internalization process—from fulfilling basic psychological needs to enhancing intrinsic motivation—before culminating in behavior. Addressing this chain mediation mechanism is crucial for developing targeted interventions for university students. Therefore, this study proposes the following research hypotheses (Figure 1):

**FIGURE 1:**
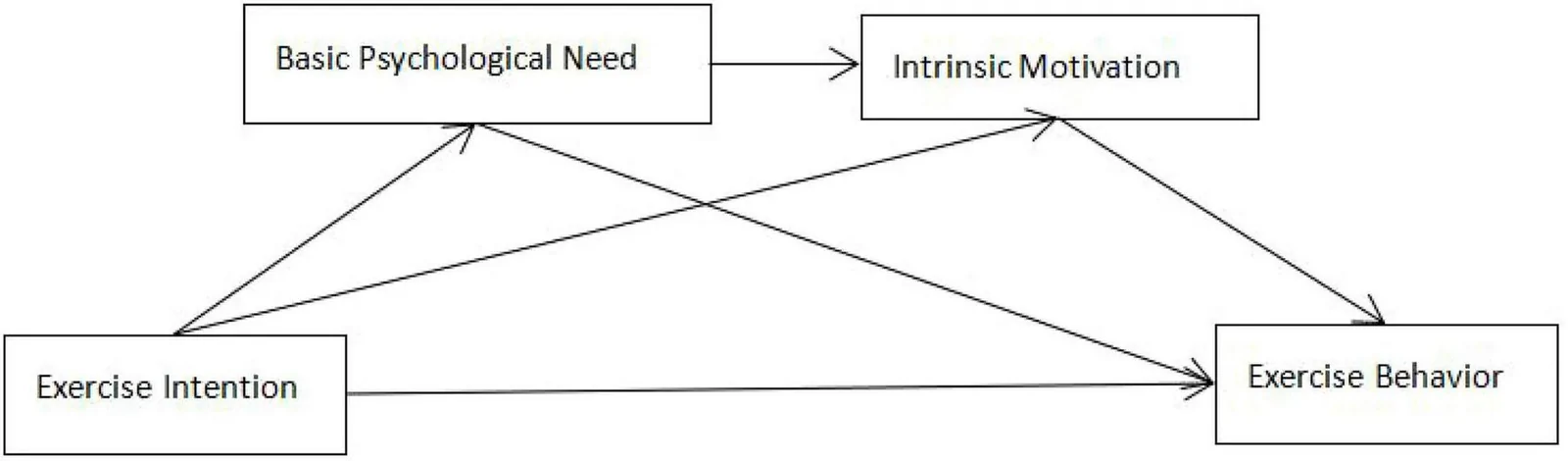
Research hypothesis model.

Hypothesis 1: University students’ exercise intentions, basic psychological needs, and intrinsic motivation are significantly positively associated with exercise behavior

Hypothesis 2: Exercise intention is linked to exercise behavior through a chained mediating mechanism involving basic psychological needs and intrinsic motivation.

## 2 Research methods

### 2.1 Data collection

This study employed convenience sampling to recruit participants from a university in Liaoning Province. A total of 1,020 students voluntarily participated in the study. Participants received an online link directing them to complete the survey, which included an informed consent form and the measurement tools used in this study. They were assured of anonymity and informed that participation was voluntary and that they could withdraw at any time. A total of 994 valid questionnaires were collected, resulting in a response rate of 97.45%. Among the participants, the mean age was 20.64 ± 1.35 years; there were 730 males and 264 females.

### 2.2 Measurement instruments

#### 2.2.1 Physical exercise intention questionnaire

Exercise intention reflects whether an individual currently participates in physical exercise and their propensity to do so in the future. A self-developed “Physical Exercise Intention Questionnaire” was used, consisting of 4 items scored on a 5-point scale ranging from “strongly disagree” (0) to “strongly agree” (4). The total score was used to indicate the intensity of the participants’ exercise intentions. The reliability and validity of this measurement were both acceptable: Cronbach’s α was 0.818, and McDonald’s ω was calculated at 0.855, indicating robust internal consistency. split-half reliability was 0.804, and item-total correlations ranged from 0.39 to 0.79 (*p* < 0.05); The results of confirmatory factor analysis were as follows: χ^2^/df = 5.077, GFI = 0.986, AGFI = 0.79, NFI = 0.976, NNFI = 0.979, IFI = 0.95, CFI = 0.95, RMR = 0.07, and RMSEA = 0.051. Although the χ^2^/df ratio and AGFI sit slightly outside the most stringent conventional thresholds, it is widely acknowledged in psychometrics that the χ^2^ statistic is highly sensitive to large sample sizes (*N* = 994). Given that other key relative fit indices (such as CFI, IFI, and NNFI) are all ≥ 0.95 and RMSEA is < 0.06, the instrument demonstrates an acceptable and theoretically sound structural fit for the current sample.

#### 2.2.2 Basic psychological needs satisfaction scale

The Chinese version of the Basic Psychological Needs Satisfaction Scale, revised by Liu Junsheng, was used. It consists of 9 items covering three dimensions: the need for competence, the need for autonomy, and the need for belonging. A 7-point scale was employed, ranging from “completely disagree” (1) to “completely agree” (7), with the total score indicating the degree to which the participants’ basic psychological needs were met (Liu et al., 2013). The reliability of this measurement was acceptable, with Cronbach’s α at 0.91, split-half reliability at 0.87, and item-total correlations ranging from 0.46 to 0.64 (*p* < 0.05).

#### 2.2.3 Exercise behavior regulation questionnaire

The Exercise Behavior Regulation Questionnaire-3 (EBRQ-3), revised by Wilson et al. (2006) was used. The original scale comprises six motivational dimensions; for this study, four items assessing internal motivation were selected. A five-point scale ranging from “strongly disagree” (0) to “strongly agree” (4) was employed, with the total score indicating the intensity of the participants’ internal motivation (Wilson et al., 2006). The reliability of this measurement was acceptable, with Cronbach’s α at 0.94, split-half reliability at 0.93, and item-total correlations ranging from 0.79 to 0.90 (*p* < 0.05). To confirm the structural validity of this isolated 4-item subscale within the present sample, A single-factor CFA (*N* = 994) showed that the standardized loadings of the 4 items ranged from 0.81 to 0.94, with McDonald’s ω = 0.94 and Cronbach’s α = 0.94, indicating excellent internal consistency of the scale. Since the data were in a 5-point Likert ordinal format, ML fit indices (χ^2^/df = 710.83, CFI = 0.44, RMSEA = 0.85) were severely inflated due to violations of distributional assumptions; after using the more robust ULS estimation for ordinal data, χ^2^/df ≈ 1.61, SRMR = 0.31, and the model fit was acceptable.

#### 2.2.4 Physical activity level scale

The Physical Activity Level Scale developed by Liang Deqing was used. The physical exercise score was calculated as:

Exercise score = Exercise intensity × (exercise duration – 1) × exercise frequency. Exercise intensity and frequency are scored on a 1–5 scale, while exercise duration is scored on a 0–4 scale. The total score ranges from 0 to 100. The classification criteria for exercise volume are as follows: scores below 19 indicate low exercise volume; 20–42 indicate moderate exercise volume; and 43 or above indicate high exercise volume (Liang, 1994). The test-retest reliability for this measurement was 0.81.

### 2.3 Statistical analysis

Data were analyzed using IBM SPSS 26.0 and AMOS 31.0 statistical software. Statistical analyses, including confirmatory factor analysis, correlation analysis, regression analysis, and structural equation modeling, were conducted to achieve the study’s objectives. The chain mediation effect was tested using the SPSS PROCESS macro (Model 6),with gender included as a covariate to control for potential demographic confounding effects. This method generates confidence intervals for indirect effects: if the confidence interval includes zero, the indirect effect of the mediating variable is considered insignificant.

## 3 Results

### 3.1 Common method bias test

Strict control procedures were implemented to minimize measurement bias. The measurement tools consisted of well-established scales with high reliability and validity as confirmed by relevant domestic studies. The questionnaire instructions explicitly stated, “This survey is for research purposes only,” and the administration process was conducted anonymously. The collected data were analyzed using Harman’s one-factor method to test for common method bias. Specifically, an item-level, unrotated exploratory factor analysis (principal component analysis) was conducted. A total of 20 individual items from all measurement tools were entered simultaneously into the analysis, with demographic variables excluded. A one-factor exploratory factor analysis was conducted on all items except demographic variables, with the rotation method set to “unrotated.” The results showed five factors with eigenvalues > 1, and the first factor explained 11.56% of the variance; therefore, the common method variance in this study is acceptable.

### 3.2 Test for differences

The results show that male students scored significantly higher than female students in terms of exercise intention, fulfillment of basic psychological needs, intrinsic motivation, and exercise behavior (Table 1).

**TABLE 1:** Comparison of differences in exercise intentions, basic psychological needs, intrinsic motivation, and exercise behavior by gender.

Variables	Gender	*n*	*M ± SD*	*t*	*p*
Exercise intentions	Male	730	15.54 ± 4.29	3.88	< 0.001
	Female	264	14.34 ± 4.39		
Basic psychological needs	Male	730	71.41 ± 18.63	3.02	0.003
	Female	264	67.66 ± 12.90		
Intrinsic motivation	Male	730	16.28 ± 4.83	3.38	0.001
	Female	264	15.18 ± 3.71		
Exercise behavior	Male	730	62.69 ± 37.34	5.89	< 0.001
	Female	264	47.15 ± 34.95		

### 3.3 Regression analysis

Correlation analysis revealed significant positive correlations between exercise intentions and basic psychological needs (*r* = 0.573, *p* < 0.01), as well as between intrinsic motivation and exercise behavior (*r* = 0.483, *p* < 0.01); basic psychological needs were significantly positively correlated with intrinsic motivation (*r* = 0.576, *p* < 0.01) and exercise behavior (*r* = 0.319, *p* < 0.01); and intrinsic motivation was significantly positively correlated with exercise behavior (*r* = 0.509, *p* < 0.01) (Table 2).

**TABLE 2:** Correlation analysis of exercise intentions, basic psychological needs, intrinsic motivation, and exercise behavior.

Variables	Exercise intentions	Basic psychological needs	Intrinsic motivation	Exercise intensity	Exercise duration	Exercise frequency	Exercise behavior
Exercise intentions	1	1	1	1	1	1	1
Basic psychological needs	0.573^*⁣*^						
Intrinsic motivation	0.623^*⁣*^	0.576^*⁣*^					
Exercise intensity	0.410^*⁣*^	0.298^*⁣*^	0.459^*⁣*^				
Exercise duration	0.468^*⁣*^	0.317^*⁣*^	0.492^*⁣*^	0.569^*⁣*^			
Exercise frequency	0.465^*⁣*^	0.316^*⁣*^	0.473^*⁣*^	0.526^*⁣*^	0.618^*⁣*^		
Exercise behavior	0.483^*⁣*^	0.319^*⁣*^	0.509^*⁣*^	0.795^*⁣*^	0.785^*⁣*^	0.783^*⁣*^	

^*⁣*^p < 0.01.

A multiple regression analysis was conducted with exercise behavior as the dependent variable and exercise intention, basic psychological needs, and intrinsic motivation as independent variables. The results showed that the model was statistically significant overall [*F*(3,990) = 192.555, *P* < 0.001] and explained 36.7% of the variance in exercise behavior (Tables 3, 4).

**TABLE 3:** Results of multiple regression analysis.

Variable	β	*t*	*p*	Tolerance	VIF
Exercise intentions	0.435	13.840	< 0.001	0.647	1.547
Basic psychological needs	0.065	1.515	0.130	0.344	2.909
Intrinsic motivation	0.207	5.370	< 0.001	0.430	2.324

**TABLE 4:** Model fit.

*R* ^2^	Adjusted R^2^	*F*	*P*
0.368	0.367	192.555	< 0.001

Regression coefficients indicate that exercise intention is significantly associated with exercise behavior (β = 0.435, *t* = 13.840, *p* < 0.001), and intrinsic motivation significantly predicts exercise behavior (β = 0.207, *t* = 5.370, *p* < 0.001). The predictive effect of the satisfaction of basic psychological needs was not significant (β = 0.065, *t* = 1.515, *p* = 0.130).

The results of the multicollinearity diagnosis indicated that the VIF values (1.547–2.909) for each independent variable were all below 10, suggesting that multicollinearity was not present.

### 3.4 Testing for a chain mediation effect

First, we examine the regression effects of exercise intention on basic psychological needs and intrinsic motivation. The results show that the regression effect of exercise intention on basic psychological needs is significant, explaining 35.2% of the variance; the regression effect of exercise intention on intrinsic motivation is significant, explaining 18.9% of the variance (Table 5); and the regression effect of basic psychological needs on intrinsic motivation is significant, explaining 56.9% of the variance (Table 6).

**TABLE 5:** Regression analysis of exercise intention on basic psychological needs and intrinsic motivation.

Variables	Basic psychological needs				Intrinsic motivation			
	*B*	*SE*	β	*T*	*B*	*SE*	β	*T*
Exercise intentions	2.375	0.102	0.594	23.266	0.460	0.030	0.436	15.269
*F*	541.320^***^				233.146^***^			
*R* ^2^	0.352				0.189			

^***^p < 0.01.

**TABLE 6:** Regression analysis of basic psychological needs on intrinsic motivation.

Variables	Intrinsic motivation					
	*B*	*SE*	β	*T*	*F*	*R* ^2^
Basic psychological needs	0.199	0.005	0.755	36.224	1312.179^***^	0.569

^***^p < 0.01.

Model 6 of the PROCESS SPSS plugin provided by Hayes (2013) was used, with exercise intention as the independent variable, exercise behavior as the dependent variable, and the satisfaction of basic psychological needs and intrinsic motivation as chain-mediating variables. The path coefficient results are shown in the Table 7. The chain-mediation model was tested using 5,000 Bootstrap resampling iterations. The results show that the indirect effect of the chain mediation path (exercise motivation → basic psychological needs → intrinsic motivation → exercise behavior) is 0.76, with a 95% CI of [0.385, 1.155]. Since the interval does not include 0, this indicates that the chain mediation effect is significant. Furthermore, after controlling for the mediating variables, the direct effect of the independent variable (exercise intention) on the dependent variable (exercise behavior) remained significant (effect size = 3.64, *p* < 0.01), indicating that basic psychological needs and intrinsic motivation partially mediate the relationship between exercise intention and exercise behavior (Table 7).

**TABLE 7:** Mediation effect test.

Path	Effect size	Boot SE	LLCI	ULCI
Total effect	4.72	0.22	4.279	5.162
Direct effect	3.64	0.27	3.116	4.169
Total indirect effect	1.08	0.16	0.746	1.385
Path 1	0.35	0.26	−0.168	0.855
Path 2	−0.04	0.05	−0.165	0.054
Path 3	0.76	0.19	0.385	1.155

Path 1: exercise intention → basic psychological needs → exercise behavior. Path 2: exercise intention → intrinsic motivation → exercise behavior. Path 3: exercise intention → basic psychological needs → intrinsic motivation → exercise behavior.

## 4 Discussion

### 4.1 The influence of college students’ exercise behavior

According to the results of multiple regression analysis, exercise motivation, basic psychological needs, and intrinsic motivation account for 36.7% of the variance in exercise behavior. The stronger a college student’s motivation to engage in physical exercise and the greater their desire to participate, the higher the intensity of their exercise participation, the longer the duration, the greater the frequency, the more proactive their approach during exercise, the more positive their emotional experience during exercise, and the better the exercise outcomes. These findings converge with previous literature cited in this study. Consistent with the findings of Xue (2010) and Dong and Mao (2020), our results confirm that intrinsic motivation is a strong predictor of exercise intensity and duration. Furthermore, aligning with Vlachopoulos et al. (2013), our data reiterate that the fulfillment of basic psychological needs is fundamental to driving physical exercise behavior. If an individual lacks the willingness to exercise, their resolve is weak, making them prone to procrastination, withdrawal, and half-hearted efforts, resulting in poor exercise outcomes. The fulfillment of basic psychological needs influences whether college students participate more actively in physical exercise. When students successfully complete self-selected exercise tasks while experiencing positive interpersonal interactions and a supportive atmosphere, their participation in physical exercise increases, and they are better able to maintain their exercise routines. Therefore, universities should strive to allow students to independently determine their exercise plans. After conducting an accurate and comprehensive assessment of each student’s physical capabilities, institutions should design optimal, challenging tasks—that is, exercise tasks that are autonomous, challenging, and participatory—to fully satisfy students’ sense of autonomy, competence, and belonging, thereby increasing and sustaining participation rates in physical exercise; Secondly, students should be encouraged to participate in physical exercise programs with a strong team atmosphere, emphasizing group-based activities to create a positive exercise environment. Engaging in physical exercise alongside like-minded peers fully satisfies students’ relational needs.

Increasing college students’ participation in physical exercise requires not only investment in facilities and institutional guidance but, more importantly, a focus on the students themselves to enhance their awareness of self-directed exercise and stimulate their motivation to participate. Students with stronger intrinsic motivation demonstrate greater interest in physical exercise, which increases the likelihood of voluntary participation and facilitates the maintenance of exercise habits, thereby fostering a healthy lifestyle. Therefore, in addition to prioritizing the creation of a conducive exercise environment, universities should grant students a central role, address their individual needs, and value their emotional experiences. By understanding differences in students’ physical fitness, interests, and athletic skills, institutions can help students experience a sense of autonomy, belonging, and competence during physical education. This promotes the transformation of external motivation into internal motivation, stimulates greater intrinsic motivation, and fosters a deep interest in physical exercise itself—enabling students to engage more actively in future exercise sessions.

### 4.2 Discussion on the chain of multiple mediating effects

Research confirms that in the process by which exercise intention influences exercise behavior, the chain of multiple mediating effects of basic psychological needs and intrinsic motivation is significant, with an effect size of 22.9%. The fulfillment of college students’ basic psychological needs promotes the internalization of motivation, ultimately influencing the emergence of exercise behavior. An intriguing finding in the present study is the specific pattern of mediation effects observed in Table 7. The confidence intervals for the simple mediation paths—from exercise intention through basic psychological needs to behavior (Path 1), and from exercise intention through intrinsic motivation to behavior (Path 2)—both included zero, indicating that these individual indirect effects were not significant. However, the full chain mediation (Path 3: intention→basic psychological needs→intrinsic motivation→behavior) was significant. This pattern highlights the unique value of the chain mediation model. It suggests that merely satisfying basic psychological needs or merely possessing intrinsic motivation in isolation is insufficient to bridge the gap between intention and actual exercise behavior. Instead, translating exercise intention into action requires a sequential, interdependent psychological process: a strong exercise intention first facilitates the fulfillment of basic psychological needs, which subsequently acts as a catalyst to internalize and strengthen intrinsic motivation, ultimately driving the actual occurrence of exercise behavior. Notably, our proposed sequence differs from Cuevas-Campos et al. (2020)’s structural model, which argues that needs and motivation predict exercise intention (Needs - > Motivation - > Intention). This divergence comes from different operationalizations of outcome variables: Cuevas-Campos’s research treats “intention” as the ultimate outcome, while this study takes “actual exercise behavior” as the ultimate outcome. We hold that initial cognitive intention catalyzes students to enter physical activity environments, where their basic psychological needs are satisfied, stimulating intrinsic motivation to transform fragile intention into sustained actual exercise behavior. University students’ exercise motivation is a process of continuously enhancing intrinsic motivation under the influence of a favorable external environment. Basic psychological needs promote exercise behavior through the mediating role of intrinsic motivation in physical exercise. Therefore, in the process of enhancing university students’ exercise behavior, emphasis should be placed on stimulating intrinsic motivation by fulfilling the needs for autonomy, competence, and affiliation. Autonomy needs can be met by increasing students’ autonomy in choosing physical exercise programs. Incorporating surveys on students’ exercise intentions during the design of activities such as morning exercises and extracurricular sports programs, and offering a variety of exercise options for students to choose from, facilitates the fulfillment of autonomy needs. This, in turn, enhances intrinsic motivation and promotes exercise behavior. The most distinctive feature of college life is communal living. Physical exercise settings provide excellent opportunities for interpersonal interaction. When classmates share good exercise habits and a supportive atmosphere, college students are more likely to choose and persist in physical exercise with the support of their peers. Conversely, if peers show little interest in physical exercise, rarely participate, and there is a lack of an exercise-friendly atmosphere—or if students lack the influence and support of peers and significant others, or feel neglected in social interactions—they may develop doubts about their behavior due to a low sense of belonging. This, in turn, reduces their desire to participate in extracurricular physical activities and leads to emotional detachment and apathy. Finally, the fulfillment of the need for competence should also be given due consideration. To stimulate intrinsic motivation, universities can appropriately introduce activities of moderate difficulty. When selecting extracurricular physical exercise programs, students should also challenge themselves appropriately by choosing activities they can overcome, thereby better demonstrating their capabilities during exercise.

## 5 Limitations

This study has several limitations. First, the use of convenience sampling to collect questionnaires means that the conclusions are most applicable to university student populations in mainstream Chinese universities with similar environments; caution is required when generalizing these findings to countries with different cultural backgrounds. Future research could employ stratified random sampling and collect more detailed background information to enhance the representativeness of the sample. Second, due to the inherent limitations of a cross-sectional study design, causal relationships between variables cannot be inferred. Future research should employ a longitudinal tracking design or an intervention experiment to verify the causal relationships among these variables.

## 6 Conclusion

College students’ willingness to exercise, basic psychological needs, and intrinsic motivation can predict changes in exercise behavior. Among these, willingness to exercise can directly influence exercise behavior and can also indirectly influence it through the chain mediation of basic psychological needs and intrinsic motivation. The Self-Determination Theory provides an explanation for the psychological pathways from willingness to exercise to exercise behavior.

## Data Availability

The raw data supporting the conclusions of this article will be made available by the author, without undue reservation.
